# Fasting metabolism modulates the interleukin-12/interleukin-10 cytokine axis

**DOI:** 10.1371/journal.pone.0180900

**Published:** 2017-07-24

**Authors:** Johannes J. Kovarik, Elisabeth Kernbauer, Markus A. Hölzl, Johannes Hofer, Guido A. Gualdoni, Klaus G. Schmetterer, Fitore Miftari, Yury Sobanov, Anastasia Meshcheryakova, Diana Mechtcheriakova, Nadine Witzeneder, Georg Greiner, Anna Ohradanova-Repic, Petra Waidhofer-Söllner, Marcus D. Säemann, Thomas Decker, Gerhard J. Zlabinger

**Affiliations:** 1 Institute of Immunology, Center of Pathophysiology, Infectiology and Immunology, Medical University of Vienna, Vienna, Austria; 2 Department of Internal Medicine III, Clinical Division of Nephrology and Dialysis, Medical University Vienna, Vienna, Austria; 3 Max F. Perutz Laboratories, Department of Microbiology and Immunobiology, University of Vienna, Vienna, Austria; 4 Department of Laboratory Medicine, Medical University of Vienna, Vienna, Austria; 5 Department of Pathophysiology and Allergy Research, Center of Pathophysiology, Infectiology and Immunology, Medical University of Vienna, Vienna, Austria; 6 Institute of Hygiene and Applied Immunology, Center of Pathophysiology, Infectiology and Immunology, Medical University of Vienna, Vienna, Austria; Univerzitet u Beogradu, SERBIA

## Abstract

A crucial role of cell metabolism in immune cell differentiation and function has been recently established. Growing evidence indicates that metabolic processes impact both, innate and adaptive immunity. Since a down-stream integrator of metabolic alterations, mammalian target of rapamycin (mTOR), is responsible for controlling the balance between pro-inflammatory interleukin (IL)-12 and anti-inflammatory IL-10, we investigated the effect of upstream interference using metabolic modulators on the production of pro- and anti-inflammatory cytokines. Cytokine release and protein expression in human and murine myeloid cells was assessed after *toll-like* receptor (TLR)-activation and glucose-deprivation or co-treatment with 5′-adenosine monophosphate (AMP)-activated protein kinase (AMPK) activators. Additionally, the impact of metabolic interference was analysed in an *in-vivo* mouse model. Glucose-deprivation by 2-deoxy-D-glucose (2-DG) increased the production of IL-12p40 and IL-23p19 in monocytes, but dose-dependently inhibited the release of anti-inflammatory IL-10. Similar effects have been observed using pharmacological AMPK activation. Consistently, an inhibition of the tuberous sclerosis complex-mTOR pathway was observed. In line with our *in vitro* observations, glycolysis inhibition with 2-DG showed significantly reduced bacterial burden in a Th2-prone *Listeria monocytogenes* mouse infection model. In conclusion, we showed that fasting metabolism modulates the IL-12/IL-10 cytokine balance, establishing novel targets for metabolism-based immune-modulation.

## Introduction

Considerable progress has been made in understanding the complex interdependency of immune cell function and metabolism. Depending on the stage of differentiation both, lymphocytic and myeloid cells seem to use diverse metabolic pathways to cope with varying bioenergy demands during their lifecycle. Alteration of cellular metabolism has been shown to impact particular immune cell functions, such as cell trafficking and cytokine secretion. This implies a mutual dependency of metabolism and immunity [1, 2]. Consequently, shifts in immune cell metabolism may be associated with distinct pathologies. However, this can also be envisaged as a tool to redirect unfavourable immune reactivity under pathologic conditions [3, 4]. Among other adaptive mechanisms, inflammation has evolved to maintain physiological homeostasis after microbial challenge of the host. The controlled induction of both pro- and anti-inflammatory mediators such as interleukin (IL)-12, IL-23, IL-6, tumor necrosis factor (TNF)-α, and IL-10 by myeloid cells plays a key role in effective immunity [5, 6], so that a well-coordinated inflammatory response can facilitate the resolution of infections. However, this process can also be detrimental if dysregulated [7].

Apart from its crucial role as a master regulator of cellular metabolic homeostasis, the enzyme adenosine AMPK has been shown to exert an important role in regulation of immunity, [8–10]. Importantly, AMPK controls dendritic and T-cell metabolic adaption and plays a key role in effector responses *in vivo* [11–13]. Furthermore, it has been demonstrated that AMPK regulates IL-10-mediated anti-inflammatory signaling pathways in murine macrophages [14].

Various extrinsic signals that regulate glucose and amino acid metabolism as well as bacterial stimuli converge on signaling factors of the phosphatidylinositide 3-kinase (PI3K) pathway, including Akt, 5′-adenosine monophosphate (AMP)-activated protein kinase (AMPK), and mammalian target of rapamycin (mTOR). These kinases lie at the crossroad of a complex nutrient hormonal signaling network coordinating the regulation of cell metabolism and effector mechanisms of the immune response [12, 15–17]. Recently, it has been shown that mTOR signaling is closely intertwined with the AMPK nutrient sensing pathway that is responsible for processing energy status, insulin, growth factors, and environmental cues, transmitting signals to downstream targets to effectuate both, cellular and the metabolic response [18]. Upon activation, AMPK induces, among other signaling cascades, the formation of the tuberous sclerosis complex (TSC) via phosphorylation of TSC2 and regulatory-associated protein of mTOR (Raptor) [19], which in turn inhibits phosphorylation of mTOR and its downstream targets, ribosomal protein S6 kinase (rpS6k) and 4E-binding protein 1(4E-BP1) [12, 20]. It has previously been reported that inhibition of mTOR by rapamycin in human monocytes or murine macrophages stimulated with lipopolysaccharide (LPS) enhances the production of IL-12 and IL-23, whereas IL-10 is blocked [21–23]. In order to further elucidate the impact of upstream regulation of mTOR signaling on its cytokine modulating effect the present study was aimed at investigating whether metabolic interference by mimicking fasting metabolism via AMPK activation could reproduce the effect of mTOR inhibition on cytokine induction in innate immune cells.

The results show that in human and mouse monocytes, glucose-deprivation with 2-deoxy-D-glucose (2-DG) as well as specific AMPK activators bring about effects similar to mTOR inhibition leading to consistent inhibition of IL-10 production. Furthermore, 2-DG was also able to reproduce the effect of rapamycin in a Listeria infection model leading to profound reduction in bacterial burden.

## Material and methods

### Cell culture

The study was approved by the ethics committee of the Medical University of Vienna (EC number 1381/2014) and conducted according to the Declaration of Helsinki (1964, including current revisions) of the World Medical Association. After obtaining informed consent of study participants, human peripheral blood mononuclear cells (PBMCs) were isolated as previously described [24]. Monocytes were isolated from PBMCs by magnetic-activated cell sorting using CD14^+^ microbeads (Miltenyi Biotech, Bergisch Gladbach, Germany) and cultured in RPMI 1640 medium, supplemented with 2 mM L-glutamine, 100 μg/ml streptomycin, 100 U/ml penicillin, and 10% fetal calf serum (FCS; PAA, Pasching, Austria). Bone marrow-derived cells from BALB/c mice were isolated and grown as previously described [25]. Briefly, bone marrow was isolated from the femur of 6 to 8-week-old mice. For differentiation of bone marrow-derived macrophages, cells were grown in Dulbecco's modified Eagle medium (Gibco, Invitrogen, Carlsbad, CA) supplemented with 10% FCS (Gibco) and L-cell-derived CSF-1 as previously described [25]. The cultures contained >99% F4/80+ cells.

In order to test the impact on macrophage polarization human blood monocytes of healthy donors were differentiated to macrophages and activated as previously described [26]. Briefly, 7d Mϕ differentiation was induced by 50 ng/ml M-CSF. Subsequent activation by 100 ng/ml LPS plus 25 ng/ml IFN-γ was conducted in order to obtain M1 Mϕ. In order to assess effects of 2-DG we performed the experiments in presence and absence of 5mM 2-DG. Similarly, we treated M-CSF Mϕ with 20 ng/ml IL-4 for 48 h with and without 5mM 2-DG to investigate effects of 2-DG on M2 Mϕ induction. Control M-CSF Mϕ with medium alone or 2-DG treatment alone without further bacterial stimulation or addition of cytokines was conducted (M0 Mϕ). The impact of 2-DG treatment was investigating by testing both alterations in cell surface staining of characteristic markers as well as cytokine release as indicated.

### Measurement of cytokine production

Monocytes (1 × 10^6^ cells) were pre-treated for 90 min with 2-DG or rapamycin at the indicated concentrations and then stimulated with 100 ng/ml LPS or 10^7^ *Listeria monocytogenes (L*.*m*.*)* in 24-well plates. Cell-free supernatant was collected after 20 hours. Human as well as murine cytokines (IL-12p40, IL-23p19, IL-12p70, IL-1β, IL-10 TNF-α, IL-6 as indicated) were measured by Luminex testing using specific matched-pair antibodies and recombinant cytokines as standards (Merck Millipore, Billerica, MA).

### Flow cytometry

Human macrophages were detached using ice-cold 1.5 mM EDTA in Hank’s balanced salt solution. Cells were washed with precooled staining buffer (PBS containing 1% BSA and 0.02% NaN_3_) and incubated on ice for 30 min with 4.8 mg/ml human IgG (Beriglobin P; CSL Behring, King of Prussia, PA) to prevent nonspecific binding of the mAbs to Fc receptors. Then, antibody-fluorochrome conjugates with appropriate isotype controls were added. Cells were incubated for 30 min on ice and then washed with the staining buffer. Samples were analyzed on a LSRII flow cytometer (BD Biosciences) and the data were further processed with the FloJo software (Treestar, Ashland, OR). Living cells were gated according to their forward- and side-scatter characteristics and dead cells were excluded using DAPI or 7-aminoactinomycin D (Sigma-Aldrich). Cells were scored positively that had a higher fluorescence than the cut-off of 0.5% of the isotype control mAbs. In graphs, geometric mean of fluorescence corrected for background staining using matched isotype control mAb is shown.

### Analysis of mTOR signaling

Monocytes (1 × 10^7^ cells) were treated and stimulated as indicated. Extract preparation and western blotting was performed as previously described [27]. Monoclonal antibodies against p-S6RP (Ser 240/244) and GAPDH were obtained from Cell Signaling Technology, Danvers, MA.

### Real-time polymerase chain reaction analysis

Monocytes (2 × 10^6^ cells) were pre-treated for 90 min with 3 mM 2-DG and then stimulated with 100 ng/ml LPS for 4 or 8 h or left untreated. Viability assays with different concentrations of 2-DG showed that >90% cells were viable at concentrations up to 3 mM. To determine the mRNA levels of *IL-10*, *IL-12B(p40)* and *IL-23A(p19)*, total RNA from monocytes was extracted using Trizol reagent (Invitrogen, Carlsbad, CA) according to the manufacturer protocol. cDNA was generated by Superscript II (Invitrogen) and real-time polymerase chain reaction was performed using ABI 7900HT thermocycler (Applied Biosystems by Thermo Fisher Scientific) with SDS 2.3 software (Applied Biosystems). Primers were self-designed using Primer Express 3.0 software (Applied Biosystems) and validated using the Human Total RNA Master Panel (Takara, Clontech Laboratories Inc., Mountain View, CA) as previously described [28]. Primer sequences are summarized in S1 Table. For relative quantification, data were analysed by the ΔΔCT method using SDS 2.3 software; target gene expression levels were normalized to the average of housekeeping genes, *EEF1A1* and *RPLP0* and are shown relative to the LPS-treated cells.

### Animals

Female, 5- to 8-week-old BALB/c mice were purchased from Charles River Laboratories (Massachusetts, US) and maintained under specific pathogen-free conditions. Animal experiments were approved by the University of Veterinary Medicine Vienna institutional ethics committee and carried out in accordance with protocols approved by the Austrian law (BMWF-68.205/0204-C/GT/2007, BMWF-68.205/0210-II/10B/2009, BMWF-68.205/0243-II/3B/2011) as well as national and international guidelines for laboratory animal care.

A scoring-system for assessment of distress of the animals was established before infection experiments were started. Based on these guidelines general condition and behavior of the animals was controlled by well-educated and trained staff (participants of FELASA B training courses). Depending on the progress of the disease, animals were monitored every 3–4 hours during the “day-phase” (7:00 am to 7:00 pm). In order not to disturb the circadian rhythm of the animals, there was no monitoring after 7:00 pm. Humane endpoint by cervical dislocation was conducted if death of the animals during the following hours was to be expected. Animal husbandry and experimentation was performed under the national laws (Federal Ministry for Science and Research, Vienna, Austria) and ethics committees of the University of Veterinary Medicine Vienna and according to the guidelines of FELASA which match that of ARRIVE.

### Listeria monocytogenes *(L*.*m*.*)* infection model

2-DG (Sigma-Aldrich, Vienna, AT) was dissolved in phosphate-buffered saline (PBS) and stock solutions were diluted in 100 μl PBS for injection. *L*.*m*. serovar 1/2 A was cultured in brain-heart infusion broth. Female, BALB/c mice (7- to 8-week-old) were injected intraperitoneally with 250 mg/kg/day 2-DG. Daily treatment with 2-DG or PBS alone was initiated from 3 days before logarithmically dividing *L*.*m*. were injected intraperitoneally and continued until the end of the experiment.

### Determination of bacterial load

Viable bacteria were enumerated in liver and spleen homogenates by plating serial dilutions on Oxford Listeria selective agar plates (Merck, Darmstadt, Germany).

### Histology and immunohistochemistry

Liver and spleen of mice were removed on day 3, fixed with 4.5% phosphate-buffered formaldehyde, and embedded in paraffin. Sections (4-μm thick) were cut and stained with hematoxylin and eosin. Mouse macrophages were detected by immunohistochemistry using rat anti-mouse F4/80 antibodies (Clone A3-1, AbDSerotec, Oxford, UK), Vectastain Elite ABC Complex (Vector 30 Ingold Road Burlingame, CA) was applied after the first antibody, and the nucleus was visualised by counterstaining the sections with haematoxylin. TissueFAXS (TissueGnostics, Vienna, Austria), a fully automated microscopy-based tissue analysis platform with a 20x/0.5 objective (EC Plan-NeoFluar, Zeiss, Jena, Germany) was used to obtain stained specimens. Quantitative analysis of F4/80-positive cells was performed using HistoQuest software (TissueGnostics) in four separate, 0.5-mm^2^ regions per specimen. The calculation algorithm used was as previously established for CD68-positive Kupffer cells [29].

### Statistics

SPSS 19 (SPSS Inc., 2010, Chicago, IL) and GraphPad Prism® software (GraphPad Software Inc, La Jolla, CA) were used for statistical analyses. For Student's *t*-tests, *P*-values ≤ 0.05 were considered significant. For group comparisons regarding cytokine production under different culture conditions, absolute cytokine levels have been used. For analysis of immunohistological staining, the staining-derived values were log2 transformed to achieve normal distribution; group comparison was done using the one-way ANOVA; all p values given as two-sided and p ≤ 0.05 was considered statistically significant.

## Results

### 2-DG skews cytokine production towards a pro-inflammatory state

**Fig 1:**
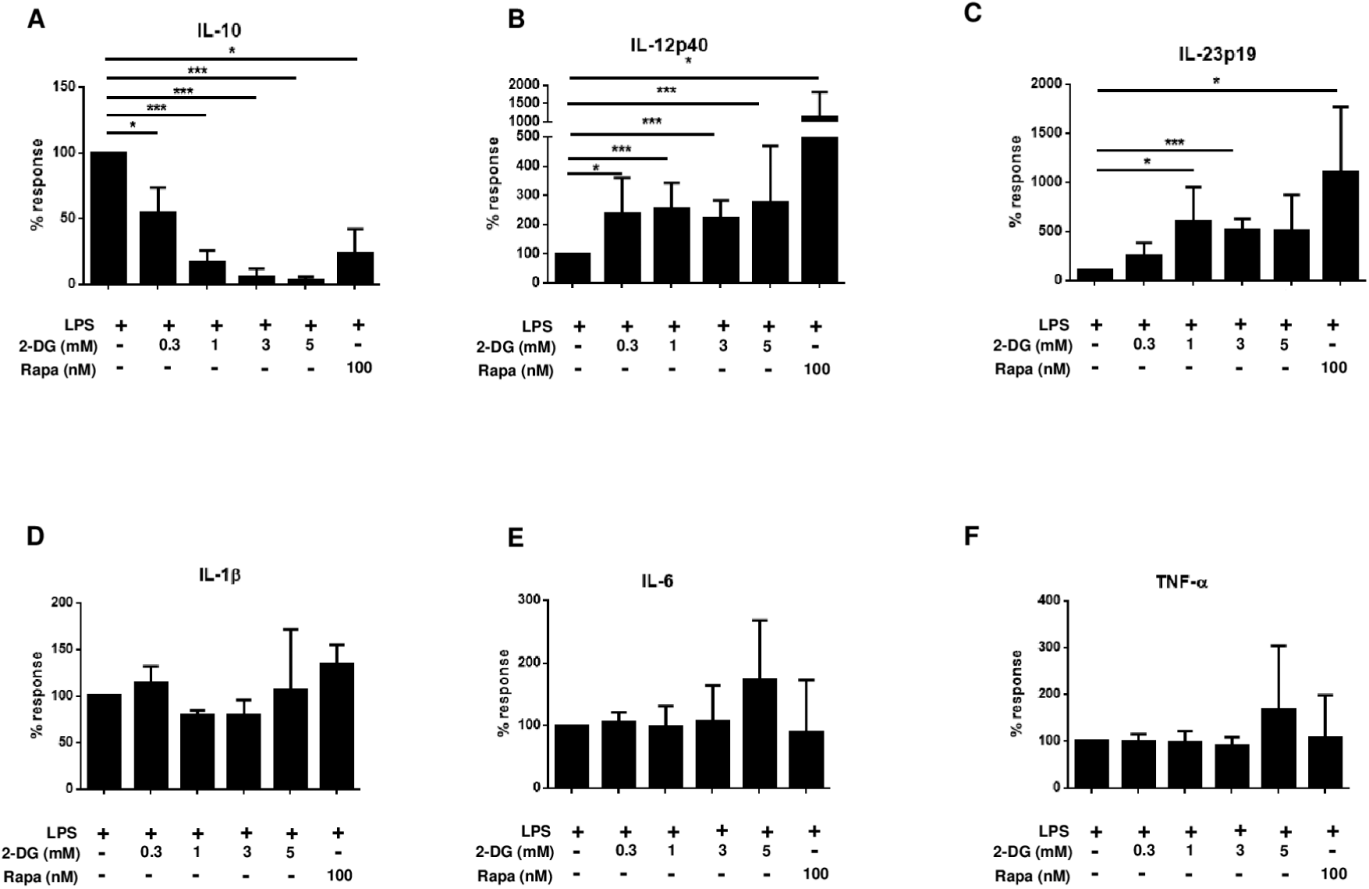
2-DG differentially modulates pro- and anti-inflammatory cytokine secretion in human monocytes. Human monocytes were preincubated for 90 minutes with different concentrations of 2-DG or medium and then stimulated with LPS. Secretion of IL-10 (a) and IL-12p40 (b) IL-23p19 (c), IL-1β (d), IL-6 (e) TNF-α (f) was determined from 18–24 hr culture supernatants by ELISA. Pretreatment of monocytes with rapamycin (100 nM) served as control. Data are representative of 5 independent experiments and presented as % response ± SD. In unstimulated cultures cytokines were undetectable. 2-DG treatment alone induced no significant cytokine production: IL-10: 2 times below detection level, 3 times ≤ 1.4 pg/mL, IL-12p40: 4 times below detection level, 1x 3.8 pg/mL, IL-23p19: 5 times below detection level, IL-1β: 2 times below detection level, 3 times ≤ 22.2 pg/mL, IL-6: 5 times below 11.6 pg/mL, TNF-α 1x below detection level 4 times ≤ 2.8 pg/mL; Mean cytokine levels after LPS stimulation in the absence of 2-DG were: IL-10, 904 ± 1096 pg/mL; IL-12p40, 535 ± 509 pg/mL; IL-23p19, 136 ± 152 pg/mL; IL-1β, 9471 ± 10692 pg/mL; IL-6, 1608 ± 414 pg/mL; TNF-α, 1596 ± 642 pg/mL. *p ≤ 0.05, ***p ≤ 0.005.

To define the potential role of metabolic interference through inhibition of glucose utilization in the regulation of pro- versus anti-inflammatory cytokines, we assessed the impact of 2-DG on cytokine induction in human monocytes after toll-like receptor (TLR)-4 engagement. Treatment of monocytes with 2-DG led to a significant dose-dependent suppression of IL-10 (Fig 1A), while pro-inflammatory IL-12p40 and IL-23p19 were upregulated (Fig 1B and 1C). Other cytokines such as IL-1β, IL-6, and TNF-α, were not altered significantly by 2-DG treatment (Fig 1D–1F).

**Fig 2:**
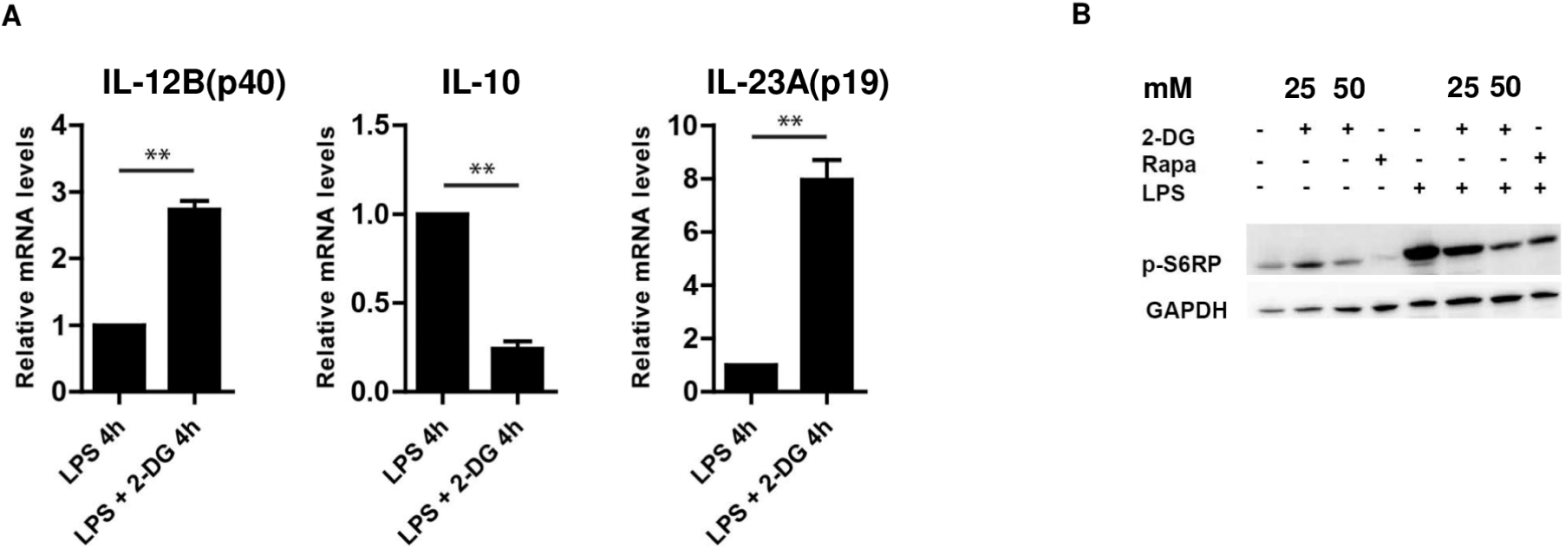
Modulation of inflammatory mediators by 2-DG at the transcriptional level and its impact of 2-DG on mTOR signaling in human monocytes. mRNA levels of IL-10, IL-12B(p40), IL-23A(p19) in human monocytes were assessed by real-time PCR analysis. Monocytes were pre-treated with 2-DG for 90 minutes or medium and then stimulated with LPS for 4 hr. Expression levels were normalized to house keeping genes and shown relative to LPS-stimulated cells. Data are displayed as means ± SD of two independent experiments. Significance was assessed by two-tailed t test; ** p ≤ 0.01 (a). Monocytes were preincubated for 15 minutes with respective mM doses of 2-DG, rapamycin (100 nM) or medium as indicated and then stimulated with LPS. Whole cell lysates were analyzed by immunoblotting using specific antibodies against phospho-S6RP (b)**.** Data are representative of 2 independent experiments.

We further evaluated whether the modulatory effect of 2-DG on cytokine production in LPS-activated human monocytes was due to transcriptional regulation. Consistent with the results of cytokine secretion, decreased mRNA expression of IL-10 with increased expression of IL-12B (p40) and IL-23A (p19) was observed in LPS-stimulated and 2-DG-co-treated cells (Fig 2A). Next, we assessed the effect of glucose-deprivation on mTOR signaling. Treatment with 2-DG of LPS-stimulated monocytes led to a dose-dependent inhibition of the mTOR downstream target p-S6RP, which was similar to that seen after rapamycin treatment (Fig 2B).

### Real-time metabolic profiling of human monocytes after injection of 2-DG

To evaluate the effect of 2-DG on metabolism of human monocytes, we performed real-time measurement of Oxygen consumption rate (OCR) and extracellular acidification rate (ECAR) on Seahorse XF24 analyzer. As described treatment with LPS dramatically increased ECAR [30, 31]. Injection of 2-DG downregulated this effect confirming the specificity of 2-DG on anaerobic glycolysis. In contrast, mitochondrial respiration, as measured by OCAR, was not affected neither by LPS or 2-DG (S1 Fig).

### Impact of 2-DG on macrophage polarization

**Fig 3:**
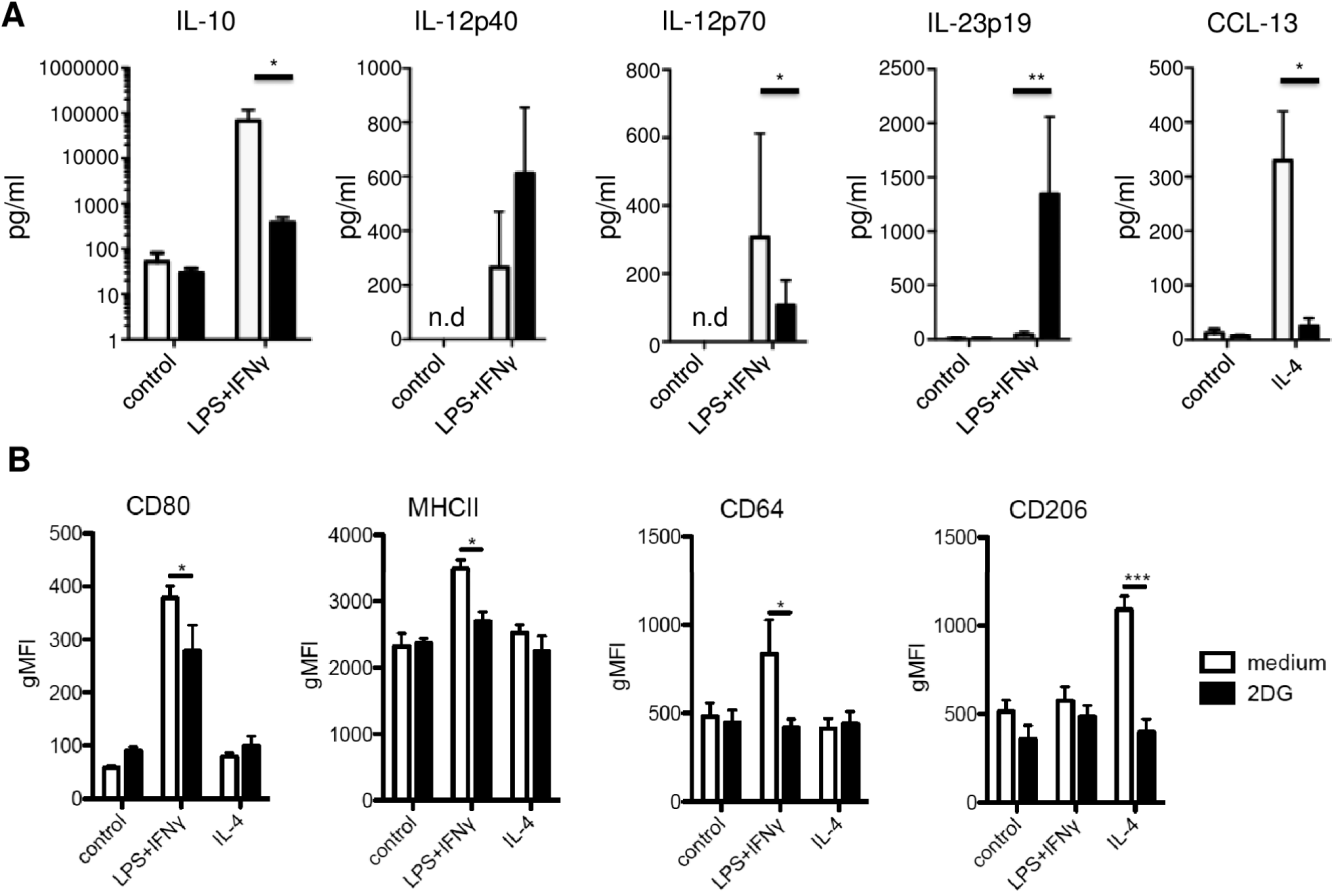
Impact of 2-DG on macrophage polarization. Human macrophages were differentiated for 7 d with M-CSF and then activated for 1 d with the indicated stimuli. (A) Secretion of IL-10, IL-12p40, IL-12p70, IL-23p19 and CCL-13 was determined from 20 hr culture supernatants by Luminex analysis. Data are representative of 3 independent experiments and presented as mean ± SD. (B) Surface expression of macrophage markers was measured by flow cytometry. Data are representative of 3 independent experiments and presented as mean ± SD. n.d. not detectable; *p≤0.05, **p≤0.01, ***p≤0.001.

In order to further analyse the impact of metabolic interference by 2-DG on myeloid cells in relation to their state of differentiation cytokine release as well as cell surface expression of characteristic markers was investigated after applying M1/M2 polarization conditions. Similar to the results obtained when testing freshly isolated human monocytes IL-10 was significantly inhibited whereas IL-12p40 and IL-23 generation was profoundly increased. Interestingly, in contrast to IL12p40, the release of IL-12 p70 was significantly inhibited. (Fig 3A). For IL-6, IL-1β and TNF-α no significant changes have been observed (data not shown). Under M2 polarization conditions (stimulation of M0 macrophages by IL-4) both CCL-13 (Fig 3A) and CCL-22 (data not shown) release was significantly inhibited.

In addition to the impact on cytokine production also surface expression of characteristic markers was investigated under M1 and M2 polarizing conditions. For all surface markers an inhibitory effect could be demonstrated (Fig 3B). These results indicate that the observed effect on cytokine production does not seem to be due to a general effect on macrophage polarization but rather be selective on the induction of particular cytokines.

### Impact of specific AMPK activators on IL-12/IL-10 induction

**Fig 4:**
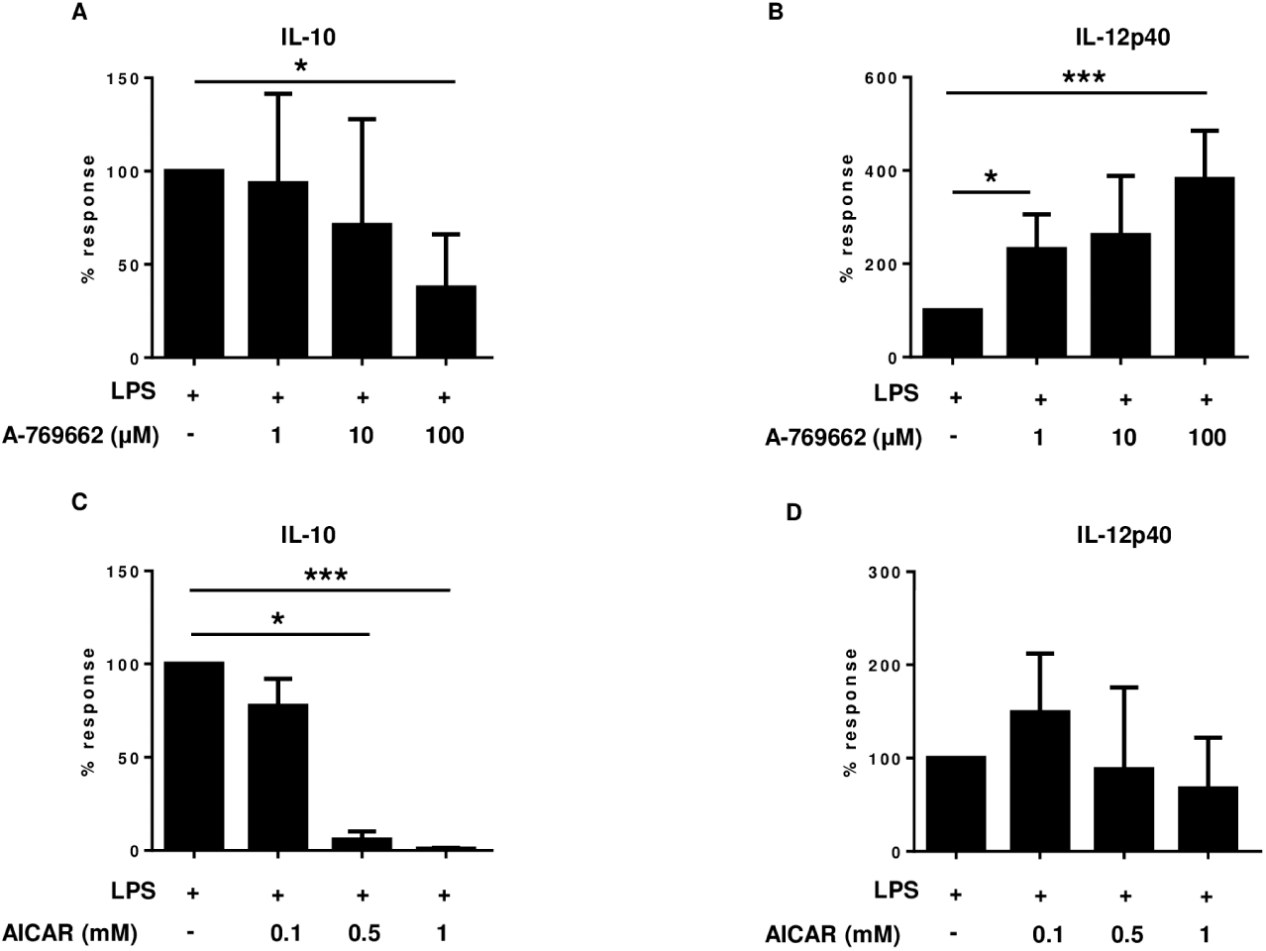
Impact of AMPK activators on IL-12p40/IL-10 induction. Human monocytes were preincubated for 90 minutes with different concentrations of A-769662 (a, b) or AICAR (c, d) or medium and then stimulated with LPS. Secretion of IL-10 (a, c) and IL-12p40 (b, d) was determined from 20 hr culture supernatants by ELISA. Data are representative of 3–5 independent experiments and presented as % response ± SD. A-769662 treatment alone induced no significant cytokine production: IL-10: 4 times below detection level, IL-12p40: 3 times below detection level 1x 1.8 pg/mL, Similarly, AICAR treatment alone induced no significant cytokine production: IL-10: 3x ≤ 3.1 pg/mL, IL-12p40: 3 times below detection level. *p ≤ 0.05, ***p ≤ 0.01.

Because of cellular energy impairment 2-DG is able to activate AMPK and AMPK activation generally is due to falling energy status and thus being indicative for fasting metabolism, we were interested in the impact of other AMPK activators in the regulation of pro- versus anti-inflammatory cytokine production. We, thus, assessed the potential of the allosteric AMPK activator, A-769662 and the AMP mimicking compound 5-aminoimidazole-4-carboxamide ribonucleotide (AICAR) to modulate the IL-12/IL-10 cytokine axis. Co-treatment of LPS-stimulated monocytes with A-769662 led to a dose-dependent suppression of IL-10 and up-regulation of IL-12p40 (Fig 4A and 4B). Identical treatment of cell cultures with AICAR also significantly inhibited IL-10 production. However, IL-12p40 induction was only marginally enhanced. Similar to 2-DG, no significant effect was seen on IL-6 and TNF-α release after co-treatment with AICAR or A-769662 (S2 Fig).

### Cytokine profile in Listeria monocytogenes *(L*.*m)* stimulated bone marrow-derived macrophages (BMDMs) from BALB/c mice

**Fig 5:**
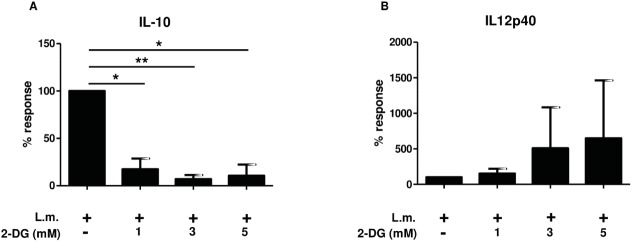
Modulation of cytokine production by 2-DG in murine BMDMs. BMDMs from BALB/c mice were cultured in 6 well plates and treated with 1, 3, and 5 mM 2-DG and *L*.*m*. as indicated. IL-10 (a) and IL-12p40 (b) were determined from 20 hour culture supernatants by ELISA. *p ≤ 0.05, **p ≤ 0.01 *L*.*m*. *Listeria monocytogenes*. 2-DG treatment alone induced the following cytokine levels: IL-10: 4 times ≤ 84.5 pg/mL, IL12p40: 4 times ≤ 5.1 pg/mL.

To validate the observed differential effect on IL-12p40 and IL-10 cytokine production *in vivo*, we first assessed the impact of 2-DG treatment on cytokine release by *in vitro* cultured, bone marrow-derived macrophages (BMDMs) from BALB/c mice. We found that similar to human monocytes, BMDMs differentially regulated IL-12 and IL-10 release upon *Listeria monocytogenes (L*.*m*.*)* challenge and 2-DG treatment *in vitro* (Fig 5A and 5B) showing a highly significant and dose-dependent reduction in IL-10 secretion after co-incubation with 2-DG (Fig 5A) with an increase in IL-12p40 secretion (Fig 5B).

### 2-DG reduces bacterial burden and granulomatous lesions in liver and spleen of *L*.*m*.-challenged BALB/c mice

To further evaluate the physiological relevance of the altered cytokine production mediated by 2-DG, we tested the effect of 2-DG in a well-established mouse infection model. BALB/c mice show an increased susceptibility to *L*.*m*. infection, which results from an inherent inability of these mice to produce sufficient amounts of IL-12, interferon-γ, and IL-6 while producing massive amounts of IL-10 [32–34] along with a Th2-skewed cytokine profile.

**Fig 6:**
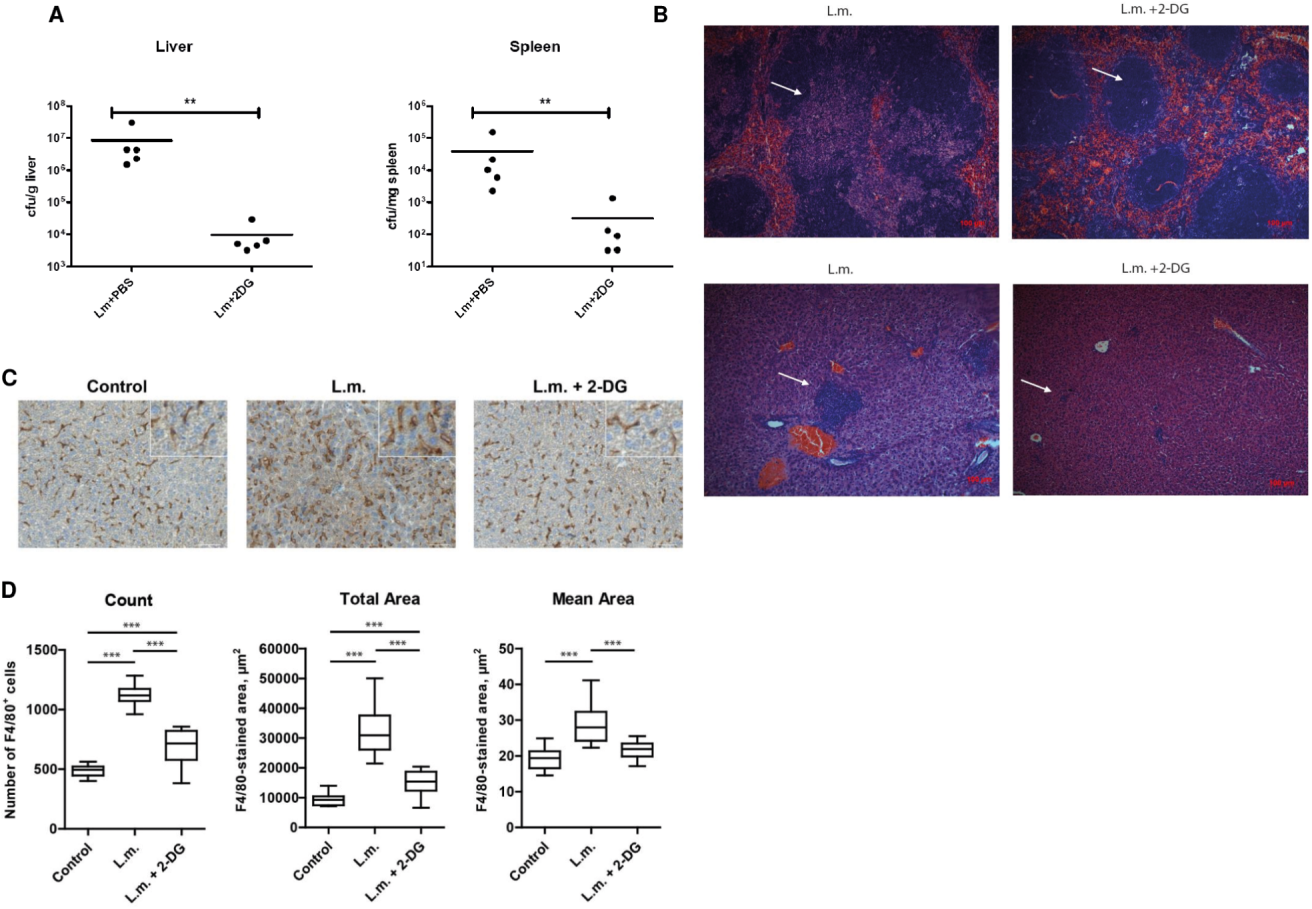
2-DG protects mice from Listeria monocytogenes *(L*.*m*.*)* infection. BALB/c mice (n = 5) were pretreated i.p. with 2-DG or PBS for 3 days and then challenged with 5 x 10^4^ *L*.*m*. On day 3 after infection the number of bacteria in the spleen and the liver were determined (a). Liver histomorphology was analyzed by H&E staining on day 3 after infection (b). Granulomatous lesions are indicated (by arrows). **p ≤ 0.005. (c) Livers (n = 3 per group) were analyzed by immunohistochemical staining of F4/80 on day 3 post infection. Representative images from each group of mice are shown (brown color, F4/80 staining; blue color, nuclear counterstaining with haematoxylin). Scale bar: 50 μm. Inserts: the high-power views. (d) Quantitative analysis of F4/80 staining in the liver specimens using HistoQuest software. The box-plot analyses represent the number of F4/80-positive cells (designated as “Count”) and also the total and mean areas of F4/80-positive cells (designated as “Total Area” and “Mean Area”, respectively); the total area values reflect both the magnitude and morphology/size of positive cells, while the mean area values reflect predominantly the morphology/size of positive cells. ***p ≤ 0.001; only significant p values are shown.

Since previous data showed *in vivo* cytokine modulating effects of 2-DG at a dose of 250 mg/kg in response to bacterial stimulation in mice [35], 2-DG was administered intraperitoneally at this dose to BALB/c mice for 3 days, followed by challenging them with *L*.*m*. infection and subsequent daily injections of 2-DG. Mice treated with 2-DG showed significantly reduced bacterial burden in the liver and the spleen at 3 days after infection as compared to mice not treated with 2-DG (Fig 6A). Additionally, mice treated with 2-DG showed reductions in the number and size of granulomatous lesions in the liver (Fig 6B), significant reduction in the number of F4/80-positive liver macrophages, as well as preservation of morphology (Fig 6C).

## Discussion

The mTOR pathway has been identified as a crucial regulator of TLR signaling in human monocytes, where direct inhibition of mTOR in LPS-activated human monocytes stimulates the production of IL-12 and IL-23 while inhibiting IL-10 [22]. We here evaluated the role of upstream interference through induction of a fasting metabolism by glucoprivation or specific AMPK activation in this process. To our knowledge, this is the first report where, similar to rapamycin, 2-DG efficiently potentiated the production of IL-12p40 and IL-23p19 in monocytes, while inhibiting anti-inflammatory IL-10. 2-DG, a glucose analogue, is phosphorylated by hexokinase to form 2-DG-6-phosphate that cannot be further metabolized and then acts as a competitive hexokinase inhibitor, thereby reducing glucose utilization and leading to impairment of cellular energy generation and also AMPK activation [36, 37] Interestingly, in a macrophage polarization model we were able to demonstrate that 2-DG was able inhibit the generation of the heterodimer IL-12p70 while showing effects similar to the monocytic system on the generation of the other cytokines tested after M1 polarization. Expression of surface marker expression characteristic for M1 and M2 polarization was inhibited which indicates that the effect of 2-DG does not seem to be due to a general effect on macrophage polarization but rather be selective on the induction of particular cytokines.

TLR-mediated changes in glycolytic metabolism have been shown previously to regulate mouse dendritic cell activation [38]. Therefore, this pathway seems to be a potential target for the control of excessive inflammation and inappropriately regulated immune responses. Interestingly, Miller *et al*. showed that, following LPS injection, 2-DG enhances the plasma levels of IL-1, IL-3, and IL-6 in female Swiss Webster mice [35], indicating a cytokine regulating potential of energy deprivation in rodents.

Dreau *et al*. demonstrated in BDF1 mice that 2-DG injections are associated with an increase in serum concentrations of TNF-α, IL-1β, and IL-6 levels in the blood of treated mice [39]. Furthermore, a recent publication by Tannahill *et al*. [40] identified succinate as a metabolite in innate immune signaling, which enhances IL-1β production during inflammation and showed that inhibition of glycolysis with 2-DG suppresses LPS-induced IL-1β, but not TNF-α production, in mouse macrophages [40]. In the present study, we observed only mild suppression of IL-1β and TNF-α at concentrations of 1–3 mM 2-DG following LPS stimulation. Although not significant, we rather observed an increase in TNF-α, IL-1β, and IL-6 secretion at higher concentrations of 2-DG (5 mM). Recently, it has also been shown that glycolysis inhibitors affect cytokine regulation as well as differentiation of human monocytes to regulatory macrophages [41, 42].

In addition to evaluating glucoprivation with 2-DG we further analysed the potential role of other AMPK activators in the regulation of pro- versus anti-inflammatory cytokines. We assessed the impact of the allosteric AMPK activator, A-769662 and also that of the AMP mimetic AICAR. Similar to treatment with 2-DG, co-treatment of LPS-stimulated monocytes with A-769662 led to a dose-dependent suppression of IL-10 and up-regulation of IL-12p40. Identical treatment of cell cultures with AICAR, an analogue of adenosine monophosphate led to a significant inhibition of IL-10, while IL-12p40 regulation, however, remained largely unaffected. Each of the activators studied has a distinct mode of AMPK-activation and a specific off-target effect profile. Therefore, the diverging results might be due to a specific mode or strength of AMPK-activation or interfering off-target effects of the compounds. Nevertheless, these results indicate that activation of AMPK, a key step upstream of mTOR inhibition, is associated with inhibition of IL-10 production, whereas induction of IL-12p40 in monocytes seems to involve additional pathways.

Of note, we also observed that at 72 hours after *Listeria* infection, BALB/c mice pre-treated with 2-DG no longer displayed signs of severe infection, indicating a prominent role of 2-DG in protection of the host against gram-positive infection by *L*. *monocytogenes*. Previously, Ogawa *et al*. demonstrated that 2-DG led to intracellular destruction of *Legionella pneumophila* in A/J mouse macrophages in a dose-dependent manner. The authors concluded that the inhibitory effect of 2-DG cannot be attributed to the inhibition of glycolysis, protein synthesis, and protein glycosylation in macrophages but rather, suggested that 2-DG promotes intracellular killing of *L*. *pneumophila* by activating some novel killing mechanism of macrophages. However, they did not investigate whether 2-DG directly interfered with the formation of legionellaphorous vacuoles or whether it augmented bacterial killing [43]. Nevertheless, Ogawa *et al*. provided the first indication that an anti-metabolic reagent (2-DG) could cause depletion of intracellular *L*. *pneumophila* in macrophages. Furthermore, Matsuda *et al*. concluded that autophagy induced by 2-DG suppresses intracellular multiplication of *L*. *pneumophila* in A/J mouse macrophages [44]. Similarly, Miller *et al*. showed that metabolic stress enhances resistance to *L*.*m*. infection in BDF_1_ mice, thereby indicating the capacity of the immune system to resist infection by certain classes of microbial pathogens [45]. The enhanced bacterial clearance *in vivo* could not be explained by 2-DG exerting a toxic effect on the bacteria which could also be confirmed by others [46] as well as in our experimental setting (unpublished). In the latter study it has been demonstrated that 2-DG induced ketogenesis was required for limiting reactive oxygen species and thus enabled adaptation to the stress of bacterial inflammation leading to protection against *L*.*m*. infection.

Regarding the impact of altered cytokine induction after 2-DG treatment, IL-12p40 can interact with a variety of different subunits like IL-12p40 itself, IL-12p35 or IL-23p19 and others [47]. IL-12p40 homodimers are considered to act as IL-12 antagonists by binding to the IL-12R [48] but are also able to induce the chemoattractant factor IL-16 in macrophages and microglia [49] and as such could contribute to the resolution of inflammation in *L*.*m*. infection in a different manner than IL-12p70. Similarly, IL-23 has been described to inhibit IL-12—dependent interferon-γ production [50] but at the same time was beneficial for protection against *L*.*m*. infection by the induction of IL-17A and guiding neutrophil trafficking as well as function [51–53]. On the other hand, Carrero et al [54] have reported that increased susceptibility to *L*.*m*. infection is at least in part due to upregulation of IL-10 on macrophages and/or dendritic cells so that inhibition of this cytokine as a consequence of metabolic interference might as well contribute to the reduction of bacterial burden and resolution of infection.

In conclusion, our results show that metabolic interference with glycolysis and also AMPK activators are able to modulate cytokine release upon bacterial stimulation, and might thus serve to evolve novel ways of metabolism-based immune-intervention.

## Supporting information

S1 Fig Effects of 2-DG on oxygen consumption rates (OCR) and extracellular acidification rates (ECAR) after LPS activation of human monocytes. Mononuclear cells isolation with Leucosep columns (Greiner Bio-one) and monocytes separation using Pan Monocyte Isolation Kit (Miltenyi Biotec) was performed according to the protocol of the manufacturer. Analysis of oxygen consumption rates (OCR) and extracellular acidification rate (ECAR) was performed using the XF24 Flux Analyzer (Seahorse Bioscience), essentially as reported previously [30, 31]. In brief, 250000 monocytes were seeded into XF 24-well cell culture microplates and allowed to recover for 1 hr. A final volume of 630 μl of buffer-free Assay Medium (Seahorse Bioscience) was added to each well. Cells were then transferred to a CO2-free incubator and maintained at 37°C for 1 hour before starting the assay. After instrument calibration, cells were transferred to the XF24 Flux Analyzer to record OCR (a) and ECAR (b) rates. The measurement protocol consisted of 3 min mixture, 2 min wait and 3 min measurement times. After 46 minutes of basal measurement, 100 ng/ml LPS and 5mM 2-DG were injected.
(TIF)

S2 Fig Impact of AMPK activators on IL-6/ TNF-α induction. Human monocytes were preincubated for 90 minutes with different concentrations of A-769662 (a, b) or AICAR (c, d) or medium and then stimulated with LPS. Secretion of IL-6 (a, c) and TNF-α (b, d) was determined from 20 hr culture supernatants by ELISA. Data are representative of 3–5 independent experiments and presented as % response ± SD. In unstimulated cultures cytokines were hardly detectable: IL-6: 4 times ≤ 87.6 pg/mL, TNF-α: 4 times ≤ 8.4 pg/mL; A-769662 treatment alone induced no significant cytokine production: IL-6: 4 times ≤ 29.8 pg/mL, TNF-α: 4 times ≤ 2.7 pg/mL, Similarly, AICAR treatment alone induced no significant cytokine production: IL-6: 3x ≤ 12.5 pg/mL, TNF-α: 3 times ≤ 0.1pg/mL.
(TIF)

S1 Table Real-time PCR primers. Gene symbol, Gene ID and sequences of forward, reverse primers and probe (when applicable) are indicated.
(PDF)

S1 Dataset Dataset. This supporting information file contains all relevant data ordered in line with the figures of the manuscript.
(XLSX)

## Abbreviations

AICAR: 5-aminoimidazole-4-carboxamide ribonucleotide
AMPK: 5′-adenosine monophosphate (AMP)-activated protein kinase
ATP: adenosine triphosphate
BMDMs: bone marrow-derived macrophages
2-DG: 2-deoxy-D-glucose
IL: interleukin
*L*.*m*.: Listeria monocytogenes
LPS: lipopolysaccharide
mTOR: mammalian target of rapamycin
mTORC1: mTOR complex 1
MAPK: mitogen-activated protein kinase
NF-κB: nuclear factor kappa B
PBMCs: peripheral blood mononuclear cells
PI3K: phosphatidylinositide 3-kinase
Raptor: regulatory-associated protein of mTOR
rpS6k: ribosomal protein S6 kinase
TSC: tuberous sclerosis complex
TNF-α: tumor necrosis factor-α
TLR: toll-like receptor
