# Supplementary figures and images for: Fasting metabolism modulates the interleukin-12/interleukin-10 cytokine axis
Source: PLoS One. 2017 Jul 24;12(7):e0180900. doi: 10.1371/journal.pone.0180900 (PMC5524343; doi:10.1371/journal.pone.0180900)

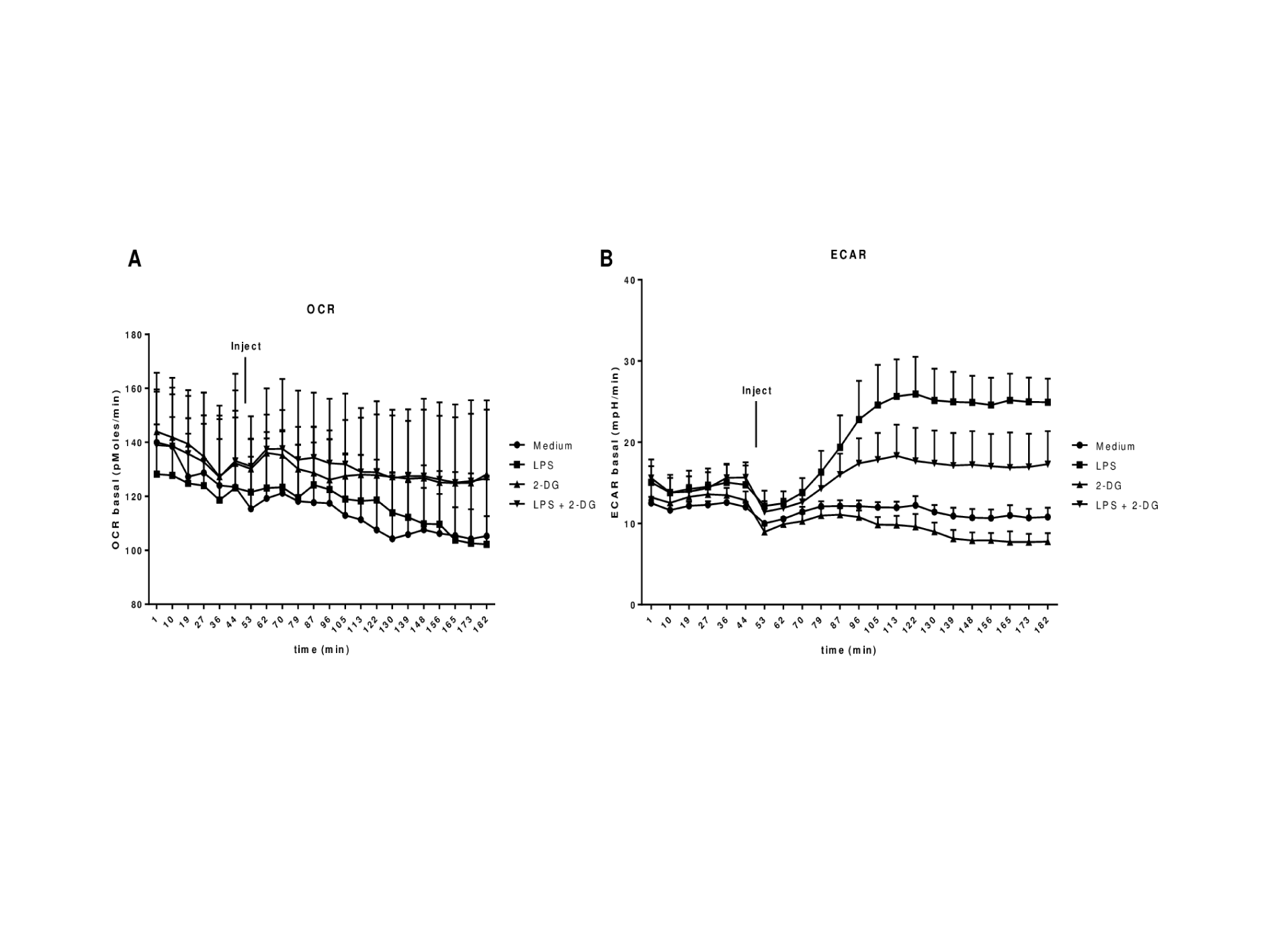

Supplement: S1 Fig — Mononuclear cells isolation with Leucosep columns (Greiner Bio-one) and monocytes separation using Pan Monocyte Isolation Kit (Miltenyi Biotec) was performed according to the protocol of the manufacturer. Analysis of oxygen consumption rates (OCR) and extracellular acidification rate (ECAR) was performed using the XF24 Flux Analyzer (Seahorse Bioscience), essentially as reported previously [30, 31]. In brief, 250000 monocytes were seeded into XF 24-well cell culture microplates and allowed to recover for 1 hr. A final volume of 630 μl of buffer-free Assay Medium (Seahorse Bioscience) was added to each well. Cells were then transferred to a CO2-free incubator and maintained at 37°C for 1 hour before starting the assay. After instrument calibration, cells were transferred to the XF24 Flux Analyzer to record OCR (a) and ECAR (b) rates. The measurement protocol consisted of 3 min mixture, 2 min wait and 3 min measurement times. After 46 minutes of basal measurement, 100 ng/ml LPS and 5mM 2-DG were injected. (TIF) [file pone.0180900.s001.tif]

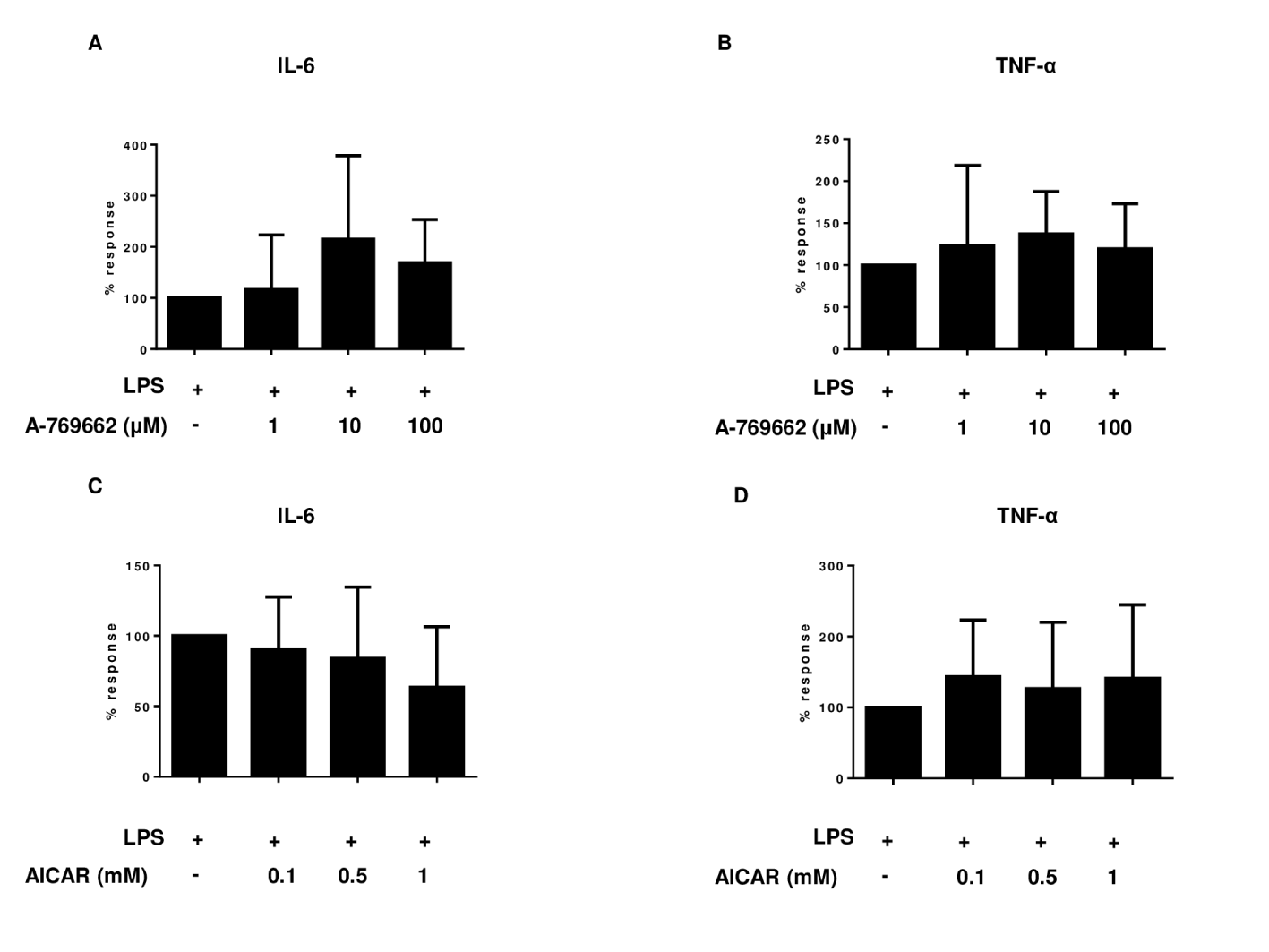

Supplement: S2 Fig — Human monocytes were preincubated for 90 minutes with different concentrations of A-769662 (a, b) or AICAR (c, d) or medium and then stimulated with LPS. Secretion of IL-6 (a, c) and TNF-α (b, d) was determined from 20 hr culture supernatants by ELISA. Data are representative of 3–5 independent experiments and presented as % response ± SD. In unstimulated cultures cytokines were hardly detectable: IL-6: 4 times ≤ 87.6 pg/mL, TNF-α: 4 times ≤ 8.4 pg/mL; A-769662 treatment alone induced no significant cytokine production: IL-6: 4 times ≤ 29.8 pg/mL, TNF-α: 4 times ≤ 2.7 pg/mL, Similarly, AICAR treatment alone induced no significant cytokine production: IL-6: 3x ≤ 12.5 pg/mL, TNF-α: 3 times ≤ 0.1pg/mL. (TIF) [file pone.0180900.s002.tif]
