# Supplementary material for: Fasting metabolism modulates the interleukin-12/interleukin-10 cytokine axis
Source: PLoS One. 2017 Jul 24;12(7):e0180900. doi: 10.1371/journal.pone.0180900 (PMC5524343; doi:10.1371/journal.pone.0180900)
Supplement: S1 Table — Gene symbol, Gene ID and sequences of forward, reverse primers and probe (when applicable) are indicated. (PDF) [file pone.0180900.s003.pdf]

| **Gene symbol** | **Gene ID** | **Forward/Reverse/Probe** |
|---|---|---|
| *IL-10* | 3586 | F: TTGCTGGAGGACTTTAAGGGTTAC<br>R: TCTGGGTCTTGGTTCTCAGCTT |
| *IL-12B(p40)* | 3593 | F: ACACATGCAAGGAGCTGAGAGA<br>R: CAGGTTCAGGAAGCCGAACA<br>FAM-CAGGTGGAGGTCAGCTGGGAGTACCC-Tamra |
| *IL-23A(p19)* | 51561 | F: AATGATGTTCCCCATATCCAGTGT<br>R: GCAAGCAGAACTGACTGTTGTCC |
| *EEF1A1* | 1915 | F: ATTACAGGGACATCTCAGGCTGAC<br>R: CATTCTTGGAGATACCAGCTTCAA |
| *RPLP0* | 6175 | F: CATCTACAACCCTGAAGTGCTTGA<br>R: CATTGCGGACACCCTCCAG |
